# Using Genomics to Decipher the Enigmatic Properties and Survival Adaptation of Candidate Phyla Radiation

**DOI:** 10.3390/microorganisms11051231

**Published:** 2023-05-07

**Authors:** Mohamad Maatouk, Jean-Marc Rolain, Fadi Bittar

**Affiliations:** 1 Aix-Marseille Université, IRD, APHM, MEPHI, 13005 Marseille, France; mohamad.maatouk@etu.univ-amu.fr (M.M.); jean-marc.rolain@univ-amu.fr (J.-M.R.); 2 IHU Méditerranée Infection, 13005 Marseille, France

**Keywords:** Candidate Phyla Radiation (CPR), genome analysis, genetic diversity, microbial interaction, adaptation

## Abstract

Microbial ecology is a critical field for understanding the composition, diversity, and functions of microorganisms in various environmental and health-related processes. The discovery of Candidate Phyla Radiation (CPR) through culture-independent methods has introduced a new division of microbes characterized by a symbiotic/parasitic lifestyle, small cell size, and small genome. Despite being poorly understood, CPRs have garnered significant attention in recent years due to their widespread detection in a variety of environmental and clinical samples. These microorganisms have been found to exhibit a high degree of genetic diversity compared to other microbes. Several studies have shed light on their potential importance in global biogeochemical cycles and their impact on various human activities. In this review, we provide a systematic overview of the discovery of CPRs. We then focus on describing how the genomic characteristics of CPRs have helped them interact with and adapt to other microbes in different ecological niches. Future works should focus on discovering the metabolic capacities of CPRs and, if possible, isolating them to obtain a better understanding of these microorganisms.

## 1. Introduction: Progress in High-Throughput Technologies Enables the Identification of New Prokaryotic Taxa

**Figure 1:**
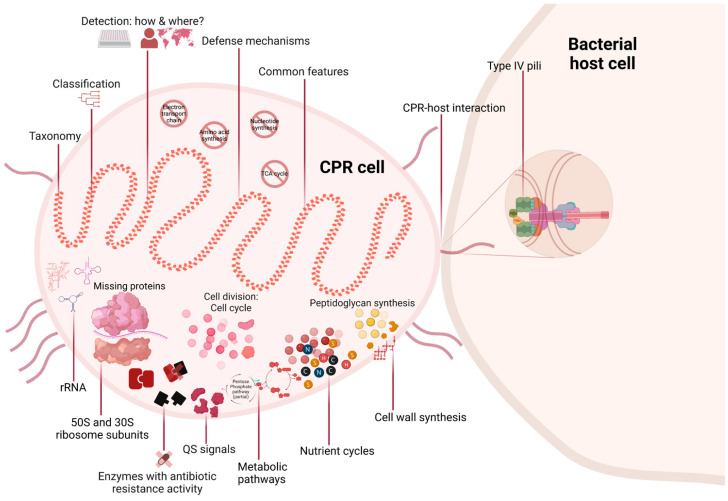
Overview of all studied axes in this literature review (created with biorender.com).

Greater in-depth knowledge of the microbial diversity of any community is crucial in order to identify its roles in various processes that affect human health and the environment. Our knowledge was previously limited to the detectable bacterial domains, of which approximately 2% are cultivable taxa [1]. Equally crucial is the hunt for unknown microbes, so-called “microbial dark matter”, for their importance in maintaining the different ecosystems [2,3]. Now, as a result of high-throughput technologies, a tsunami of new genome sequences has reshaped our understanding of the diversity of life [4]. However, these methods have revealed that the proportion of unknowns may be even higher than expected. Due to insufficient sequencing depth and the complexity of performing genome assemblies, it was initially challenging to sequence populations with less than 1% relative abundance in complex metagenomes [5]. Single-cell genomics was the solution, but the technique was hampered by an amplification bias [2,6]. However, rapid improvements in the quality of sequencing enabled the discovery of Candidate Phyla Radiation (CPR) (Figure 1) [7]. This group of prokaryotic organisms is a large evolutionary radiation whose members have not yet been fully characterized. As the name indicates, a “candidate phylum” (analogous to a “candidate division”) has no cultured representative to date, but its existence has been established through culture-independent molecular methods [8]. Notably, some CPR groups could not be detected in rRNA research due to primer mismatch, introns, or both [9,10]. However, microfluidic single-cell dispenser systems have made it possible to analyze non-culturable microbial cells, including CPRs (Figure 1) [11].

CPRs, also referred to as “ultramicrobacteria” or “nanobacteria”, have a reduced genome (around one megabase pair) and are small in size (100 nm to 300 nm), with a cell volume of 0.01 μm^3^ (Figure 1 and Table 1) [5,7,12]. This volume was revealed by cryogenic transmission electron microscope images that targeted post-0.2 mm filtrates collected from environmental samples [13]. Moreover, metagenomic and/or metabarcoding analyses have revealed that CPRs are ubiquitous in different ecosystems around the world. They have been detected in samples of drinking water, deep subsurface sediments, soil, and even in anaerobic subsurfaces where organisms from extremely ancient lineages that diverged from primitive life forms reside [4,9,14,15,16,17,18]. CPR superphyla have also been reported in clinical samples of the human microbiome (Figure 1) [19,20,21,22,23].

The CPR group is diverse, representing more than 26% of known microbial species [24]. This abundance is uncertain and depends on the type of analysis performed and the time it is carried out [7,12,25]. A dramatic increase in biological data has significantly expanded the scale of the microbial community, and new species are regularly being identified. CPRs include more than 70 phyla, with around 11,000 genomes, most of which are incomplete and considered as metagenome-assembled genomes (MAGs) in the National Center for Biotechnology Information (NCBI) database [26]. CPRs are also sometimes referred to as “Patescibacteria”, since more than 74% of their genomes belong to this superphylum or phylum [27,28]. Nevertheless, the distinction between superphylum and phylum has not yet been clearly described for this radiation. Moreover, as there is no proposed formal name for this division, the misuse of “Patescibacteria” as a synonym for CPR is confusing when addressing a superphylum/phylum particularity without generalizing it to the whole CPR group.

## 2. CPR Genomes: Discovery of a New Division Is Challenging for Evolutionists

The first sequence of CPR was detected in 1996 [29]. It was discovered by detecting a 350 bp fragment of a 16S ribosomal subunit (rRNA) of a TM7 (Torf, mittlere Schicht, clone 7, or “peat, middle layer, clone 7”) [29]. It was then inserted in a phylogenetic tree analysis. TM7 is now referred to as *Candidatus* Saccharibacteria superphylum, due to its sugar metabolism, after the first complete genome was revealed in 2007 [30].

Two years after a CPR sequence was first detected, in 1998, the 16S rRNA phylogenetic tree revealed CPR sequences as a distinct phylum independent of archaea and eukaryotes but related to bacteria (Figure 1) [31]. Due to significant divergent genetic sequences, some studies have shown the inadequacy of using 16S rRNA for CPR classification [7,32]. However, the use of other conserved coding genes, such as ribonucleotide reductase (RNR), topoisomerase IIA (Topo IIA), elongation factor 1 (EF1), DNA-dependent RNA polymerase subunit II (RNAP II), thymidylate synthase (ThyA), and DNA polymerase, has ultimately resulted in a very similar classification of CPR genomes [32,33]. Thus, a recent genomic analysis conducted by Megrian et al. showed the conserved presence of division and cell wall (dcw) genes with the necessary order (synteny) in over 1000 bacterial genomes, including CPRs [34]. Based on the concatenation of this gene cluster, phylogenetic trees have shown consistency with previous phylogenies in which dcw genes can be used as alternative markers to produce a robust phylogenetic tree (Figure 1) [34].

CPRs may have grouped together, as they share the protein profile (presence and absence) of certain protein groups in comparison to other domains of life, although they were directly branched as a deep ramification of the bacterial kingdom (Figure 1) [12,35,36,37]. Therefore, CPR cannot be regarded as a novel domain but rather as a superphylum of bacteria, since their contribution of only 1.22% to the repertoire of bacterial proteins was insufficiently significant [37]. Studies using phylogenomic trees based on single or multiple genes have shown that CPR and bacteria are two paraphyletic groups and not two distinct divisions (Figure 1 and Table 1) [36,37]. This model is currently a matter of debate. In contrast, rhizome-based classification, based on the total genome sequences and taking into consideration sequences generated by processes such as transfers, recombination, fusion, degradation, and de novo synthesis, has shown that CPR is a genetically independent division [38]. This classification also takes ORFans into account, which are genes that are specific to a certain species and have an unknown evolutionary identity. The rhizomes of CPR were mosaic and composed of sequences from heterogeneous origins, unlike the homogeneous profile of those of bacteria [38]. These differences in rhizomal profiles confirm the particularity of the historical evolution of CPR genomes.

One study in which CPR was distinguished based on protein family content suggested two possibilities regarding how CPRs have evolved [36]. The first possibility is that CPR and non-CPR bacteria could have co-evolved after emerging from the protogenote community, and CPR genomes subsequently diversified over time. The second possibility is that CPRs could have evolved from non-CPR bacteria after having undergone a severe genome reduction [36]. The latter scenario has not been supported by numerous findings, mainly since CPRs do not share genomic features with recent genome reduction organisms (Figure 1 and Table 1) [39,40]. CPRs have thus demonstrated the capacity to maintain the integrity of their genomes by having genes involved in DNA mismatch repair and homologous recombination pathways (such as Holliday junction resolvases) [9]. Moreover, CPRs exhibit active metabolic patterns that aggregate independently of another metabolically reduced symbiont (Figure 1) [7,12,24,41,42]. Therefore, the uniformly small cell size and genome of CPRs were found to be ancestral characteristics of their group rather than the result of starvation.

Through the detection of CPR sequences by metagenomic analysis, these microorganisms were identified as a new microbial discovery, the definition of which changes from one analysis to another. However, CPRs are distinct and divergent from other groups of life (Figure 1). Their taxonomy and classification represent a continuous challenge for microbiologists. Consequently, their placement within the tree of life is not yet well-established, and their evolution remains a mystery to be elucidated.

## 3. Distinct Biology of CPR Cells

### 3.1. Common and Unusual Genomic Features of CPRs

CPRs are characterized by the presence of a single copy of 16S rRNA with self-splicing introns and protein insertions that are rarely reported in classical bacteria (Figure 1 and Table 1) [7]. This has also been shown in sequences of 23S rRNA and tRNA [9,43]. Due to these intron sequences, CPRs have larger rRNA than known bacteria, but are expected to be similar in length after transcription (Table 1) [44,45]. Moreover, genomic analysis of CPR ribosomes revealed missing protein regions on their surface structure. This was also the case for CPR rRNA (23S rRNA, 5S rRNA, and 16S rRNA) (Figure 1 and Table 1) [44]. Thus, the absence of a few proteins in CPR ribosomes was revealed by genomic comparisons such as rpL30, uL1, uL30, and bL9, which are usually found in bacteria [44]. This suggests that CPR ribosomal proteins have distinctive compositions compared to those of classical bacteria. Furthermore, in some CPR genomes of different phyla (such as Parcubacteria, Saccharibacteria, Microgenomates, and Katanobacteria), a lack of rpL1, rpL9, rpL25, uL29, bL28, bL32, and bL33 has been identified [44]. Some of these ribosomal proteins are universal in bacteria and are involved in essential processes of the formation, growth, and regulation of ribosome subunits [7]. All these heterogeneities of proteins in ribosome structures point to diverse trajectories of ribosome evolution between bacteria and CPRs and across CPR lineages [44]. It is thought, therefore, that CPRs probably have analogous proteins and/or alternatives to fulfil the functions of those that are missing.

Genomes of CPR have shown fewer open reading frames (ORFs) than other bacteria, which indicates a correlation between bacterial genome size and the number of encoded proteins (Table 1) [45]. Furthermore, based on an analysis of a massive metagenomic dataset, CPR genomes were found to harbor diversity-generating retroelements (DGRs) [46]. DGRs are mobile elements that diversify DNA sequences by introducing nucleotide substitutions at specific locations, thereby creating different protein-encoding genes [47]. They were found to be abundant in microorganisms with small genomes and episymbiotic lifestyles, such as CPRs [48]. Their function expands ligand specificity for different cellular binding proteins involved in attachment with host cells [28]. This process could accelerate the evolution of CPRs to exploit potential hosts in different environments. They may be a result of a myriad of selective pressures to impose genetic hyper-variability, which is particularly advantageous for adaptation (Figure 1) [47,49,50].

A high ribosome copy number is considered to be a driver of rapid growth, suggesting that multiple rRNA gene-carrying microbes are generally more efficient in responding to resource availability (Figure 1 and Table 1) [51]. However, CPRs with a single copy of the 16S rRNA call upon multiple mechanisms in life-and-death struggles, providing prodigious levels of diversity. From splicing introns in their rRNA to DGRs in their genomes, these different genetic diversification mechanisms enable CPR cells to adapt to various environmental conditions (such as limitations on energy or nutrients).

### 3.2. Metabolic Pathways: CPRs Need to Do More with Less

Little is currently understood about the metabolic activity of CPRs, since most of their genomes are incomplete, which prevents a total/complete in silico analysis. Thus, CPRs with no pure culture to date lack isolated representatives, hence in vitro analysis of their metabolic pathways is not possible (Figure 1 and Table 1). However, CPRs are characterized by their obligate exo-symbiotic/parasitic lifestyle in which their survival depends on a host (most often anaerobic bacteria) (Table 1). This dependency is variable between CPR lineages, as they have different biosynthetic capacities. Almost all CPRs have shown the inability to autonomously synthesize nucleotides (de novo), amino acids, and membrane components (Figure 1) [7,13,15,52]. However, the analysis of five genomes of the Peregrinibacteria phylum revealed a semi-independent lifestyle toward its host [53]. They showed fuller metabolic pathways than other CPRs, with the ability to significantly synthesize cell wall components, including peptidoglycan, isoprenoids through a near-complete mevalonate pathway, and other elements [53]. However, this absence of recognized/essential processes in all CPR cells has made them polylithic in terms of metabolism and auxotrophic microorganisms (Figure 1 and Table 1).

CPRs lack the necessary pathways to synthesize lipopolysaccharide (LPS) and produce fatty acids [9,54]. They appear to lack the ability to synthesize lipid A, which is necessary for a Gram-stain-negative cell envelope [55]. However, multiple studies anticipate cell-wall-containing peptidoglycan, in which all required genes for its biosynthesis were identified (Figure 1 and Table 1) [9,53]. Hence, microscopic observations were consistent, in that CPR cells have a Gram-stain-positive-like membrane [13]. They were thus found to encode large extracellular proteins rich in cysteine, which may function in cell attachment [53]. Moreover, genomic analysis of the Saccharibacteria and Absconditabacteria genomes has revealed an interesting pattern for several metabolites, with high PM values (quantifying metric for biosynthetic capabilities) [56]. These metabolites were mostly components of the CPR cell wall [56].

Based on genome analysis, CPRs have shown an anaerobic respiratory lifestyle with metabolic patterns that match the Earth’s early anaerobic environment [57]. Most CPRs have the glycolysis Embden-Meyerhof-Parnas (EMP) and the pentose phosphate pathway (PPP), whereas downstream biosynthetic pathways were absent for the PPP [57]. Thus, they have neither an electron transport chain, nor tricarboxylic acid (TCA) cycle, nor subunits of NADH dehydrogenase (Figure 1) [57]. The absence of these essential compounds suggests a fermentation-based metabolism, especially given that CPRs have shown the synthesis of acetate, lactate, ethanol, and hydrogen as fermentation end products with missing crucial elements of respiratory pathways [57]. However, a particular CPR genome known as “*Candidatus* Parcunitrobacter nitroensis” has revealed a complete electron transfer chain. This genome contained all genes involved in the nitrogen-respiration process (hydroxylamine redox enzyme, nitrate reductase, and nitric oxide reductase) [58]. Moreover, CPR genomes have shown the presence of nitrite reductase genes (nirK), key to the denitrification process (Figure 1) [16,59]. These are responsible for the nitrogen cycle, as they reduce nitrite to nitric oxide.

Furthermore, the analysis of CPR genomes has shown different glycoside hydrolase (GH) profiles/families [60]. This diversity allows carbon processing to a wide range of carbon substrates and complex sugars present in the environment (Figure 1) [61]. This dynamic behavior of different CPR phyla has also been shown through the presence of genes such as *NiFe* hydrogenases and iron-only hydrogenases involved in sulfur cycling [57]. Thus, despite the absence of sulfur respiration genes, CPRs can effectively participate in the sulfur cycle via hydrogenase-mediated sulfur reductase activity (Figure 1) [62]. Some other genomes have shown sulfur dioxygenase (*sdo*) and sulfate reduction genes (*sat*, *cysC*, and *cysN*) [63]. Through these processes, CPR organisms can play a vital role in nutrient cycling/secondary metabolism by breaking down complex organic molecules that other microorganisms are not capable of doing.

It is important to note that, as is the case with symbiotic cells, CPRs can vary their metabolic capabilities depending on the content of the niche and on the availability of what the host offers/provides. These distinctions have been shown by the content of enzyme-encoding genes with antibiotic resistance activity in CPR genomes detected in the human microbiomes being higher than those detected in environmental samples (Figure 1) [64]. It has also been shown through the detection of an arginine deiminase system (ADS) in Saccharibacteria genomes during the niche transition from the environment to mammalian microbiomes [65].

The diversity of metabolic capacities in widespread microorganisms suggests that CPRs are key members in driving critical elements via biogeochemical cycles for the microbe community [66], especially as CPRs have been found in various environments, including soil, freshwater, and marine systems (Figure 1). However, the different genes of metabolic pathways in CPR cells may function to obtain necessary nutrients and/or amino acids that are not encoded in their genomes, to capture energy, or to protect against a particular substrate in the niche they occupy. They may also play a regulatory role in the selection and attachment to the bacterial host membrane.

## 4. Beyond the Surface: Revealing the Diversity of Interactions and Signaling Mechanisms of CPRs in Microbial Ecosystems

The study of CPR is still in its early stages, and much remains to be learned about their interactions with other microbes. These microorganisms are small but well-equipped with genes that play a role in the functioning of ecosystems including the formation of biofilm, the production of virulence factors, and antibiotic resistance.

### 4.1. Chemical Signalling

CPR cells are involved in three modes of communication to maintain their interactions with other species in the environment: quorum sensing (QS), signal presence, and attentiveness to external signals. CPR organisms have been found to use quorum sensing to coordinate the expression of their genes in influencing their hosts/neighbors (Figure 1) [67]. Chemical signaling via QS is mediated by emitting small molecules known as autoinducers. When these autoinducers reach a threshold concentration, CPR can detect them and responds by altering its gene expression, depending on the type of autoinducer.

The rich repertoire of quorum-sensing proteins in CPRs was detected using a computational in silico analysis. Charles et al. showed that these protein-coding sequences are divergent and under strong selective pressure in CPR genomes [67]. The diversity of QS signals allows a wide range of interactions/communications between CPRs and other microorganisms. Thus, CPRs were involved in the biofilm formation of their bacterial host by emitting the AI-2 QS signal [68,69,70]. This surface-associated community allows resistance to a stressful stimulus due to the creation of a dense community of CPR/host in their niche [71,72]. Moreover, the abundance of some CPR cells has been correlated with the increase of virulence factors controlled by the QS signals/receptors system [15]. Some virulence factors, such as oligopeptide transport and iron acquisition, have been detected and correlated with the presence of the CPR phylum “*Candidatus* Saccharibacteria” in periodontal mucosal disease [73]. Several studies have shown that CPR phyla are associated with a wide range of pathologies including inflammatory, mucosal, and infectious diseases [74,75,76]. These microorganisms therefore have an impact on human health, as their abundance was recently highlighted in the human microbiome [77]. Different CPR phyla have been detected in the oral microbiota and include *Candidatus* Absconditabacteria (SR1), *Candidatus* Parcubacteria (OD1), *Candidatus* Microgenomates (OP11 and OP10), *Candidatus* Gracilibacteria (GN02) and, mainly, *Candidatus* Saccharibacteria [78,79]. These and others have also been detected in the gastrointestinal tract, blood, eyes, skin, and even in the genital tracts of both genders (Figure 1) [19,20,21,22,23]. However, it remains unclear how CPR cells affect the physiology of the human microbiome. Thus, a recent study was conducted on diapause larvae of *Clanis bilineata tsingtauica* to investigate the mechanisms by which gut microbiota regulate nutrient synthesis and metabolism in edible insects [80]. The study revealed that TM7 plays a crucial role in modulating intestinal metabolites by modulating metabolic pathways, leading to an increase in linolelaidic acid (LA) and a decrease in tricosanoic acid (TA) levels via intestinal enzymes [80].

Finally, these processes of production, detection and response allow CPR cells to contribute towards the functioning of the ecosystem in which they live. Furthermore, the combination of these different communication systems may allow for a more sophisticated form of interaction to convey different types of information. It helps CPR cells to coordinate their existence with their hosts/neighbors in order to adapt to various environments.

### 4.2. Defense Mechanisms

CPRs harbor enzyme-encoding genes with antibiotic resistance (AR) activity [64]. In 2021, an exhaustive in silico analysis using a new adapted strategy of AR gene research was applied to CPR genomes (*n* = 4062; all CPR genomes available on 12 September 2020 in the NCBI database) (Figure 1). According to the NCBI Conserved Domain Database (CDD) search, these AR genes have the functional domain necessary to inactivate the antibiotic and to protect or alter its target. This analysis has revealed a rich reservoir of 30,545 AR genes, with 89 enzyme activities in all CPR superphyla (*Candidatus* Parcubacteria, *Candidatus* Microgenomates, *Candidatus* Saccharibacteria, unclassified Patescibacteria group, Candidate division WWE3 (Katanobacteria), *Candidatus* Peregrinibacteria, *Candidatus* Berkelbacteria, *Candidatus* Dojkabacteria, *Candidatus* Doudnabacteria, *Candidatus* Gracilibacteria, *Candidatus* Absconditabacteria, Candidate division Kazan-3B-28, and *Candidatus* Wirthbacteria, with an average of 7.5 AR/genome [0 to 41]) [64]. They were associated with 14 antibiotic families, the most abundant of which are glycopeptides, beta-lactams, macrolide–lincosamide–streptogramin (MLS) resistance families, tetracycline, and aminoglycoside. Moreover, the activity of five beta-lactamase sequences from CPR have been confirmed in vitro using liquid chromatography-mass spectrometry [81]. These CPR proteins have shown the ability to hydrolyze different substrates of beta-lactam (amoxicillin, ampicillin, and penicillin G), even after the addition of clavulanic acid (beta-lactamase inhibitor) [81]. Thus, some of these proteins have shown an additional RNase activity in the presence and absence of EDTA chelator and sulbactam (beta-lactamase inhibitors) [81].

However, all these enzyme-encoding genes detected in CPR genomes may have not existed for AR activity as a main function, but rather to degrade/hydrolyze different compounds in their ecosystems. Hence, a functional in silico analysis of all uncharacterized CPR MBL sequences (*n* = 3349) revealed highly divergent sequences with many protein domain functions. This recent study revealed a multifunctional activity for CPR MBL when compared to bacterial sequences, which opens possibilities for CPR cells to adapt to different environments.

Furthermore, a comparative genomic analysis of CPR in activated sludge systems has provided insights into the adaptive response of CPRs to high oxygen conditions in aeration tanks. This was revealed by significantly enriched genes for oxygen stress resistance [66]. Additionally, numerous CPR genomes were found to harbor multidrug resistance genes, including multidrug efflux pump (LfrA) and methylenomycin A resistance protein (mmr) [66].

Most CPR genomes belonging to the Patescibacteria superphylum have shown an absence of phage receptors on their cell membranes, which could help protect them against phage invasion [82]. Thus, a meta-analysis conducted by Burstein et al. showed that some Parcubacteria and Microgenomates genomes can avoid these invasive genetic elements by having viral defense mechanisms such as the CRISPR-Cas system (clustered regularly interspaced short palindromic repeats, CRISPR-associated) (Figure 1) [83]. It should be noted that these systems have rarely been found in CPR cells, but when detected they showed an unexpected diversity in CPR genomes and were significantly distinct from those of bacteria [84,85,86]. However, few CPR genomes have been reported with phage infection [87]. The infection process has also been observed via cryogenic electron microscopy [13]. Moreover, analysis of CPR genomes has shown other phage defense systems such as the DISARM (defense island system associated with restriction–modification), and the BREX (Bacteriophage Exclusion) system [88]. A genomic analysis for Saccharibacteria genomes has shown the presence of restriction–modification systems to defend against foreign DNA [89]. In addition, it was shown that CPR cells can be resistant to heavy metals (cobalt–zinc–cadmium, copper, and arsenic) present in their habitat as well as resistant to ultraviolet light (Figure 1) [88].

### 4.3. Contact-Dependent Signaling Involves Direct Cell-to-Cell Interactions

One essential feature of CPRs is their obligate attachment to a bacterial host [77,90]. To date, this episymbiotic lifestyle of CPRs continues to be underexploited, although a few studies have shown a key role of type IV pili (T4P) (Figure 1) [13]. Type IV pili enable CPR cells to perform twitch-like movements that are essential for host recognition and cell adhesion in parasitic association [77]. Generally, T4P are ubiquitous in CPRs with different functions involved in processes such as protein secretion, and they exchange supplies (metabolites) with their host (Figure 1) [13,91]. This physical association provides easier DNA uptake (competence) and sequence transfer (lateral transfer) between the host and CPR cells, which have been shown to harbor genes from organisms living in the same habitat [92,93]. T4P can even act as nanowires carrying an electric current between the two cells [94]. Moreover, the pilin proteins of T4P are important components of the extracellular matrix which is crucial for maximal biofilm formation. Thus, it is considered a valuable factor in virulence mechanisms when pili biosynthesis via QS is correlated with an increase in TM7 in the oral microbiota of patients with periodontitis disease [73].

## 5. Conclusions

**Figure 2:**
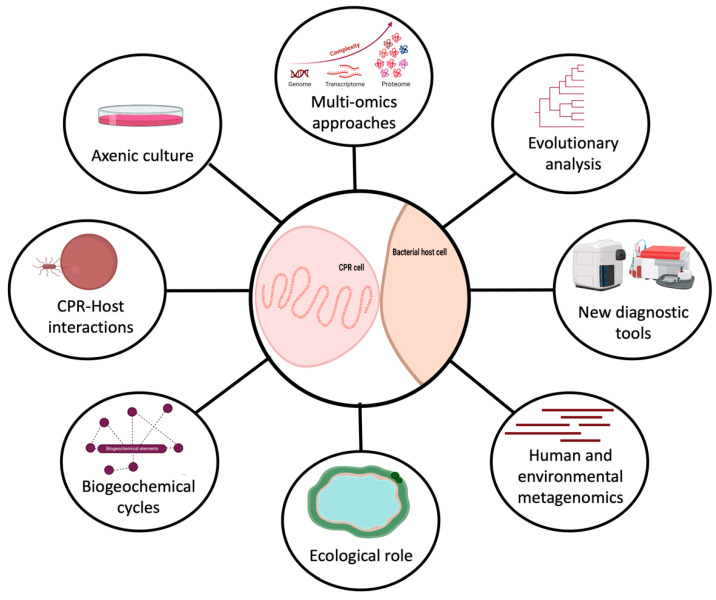
Representation of the future directions to study CPR microorganisms (created with biorender.com).

One interesting aspect of CPR organisms is that they have been found to have a high degree of genetic diversity and limited, but divergent, metabolic capacities that make them able to colonize a wide range of environments. These minimalist microbes have the potential to serve as useful model organisms for studying adaptation in various ecological niches. They are small but powerful microorganisms that can do more than expected with minimum genetic content. There is a critical need to improve techniques to enable fast and comprehensive analysis of their metabolic pathways, especially in order to access the biologically useful context that can be extracted from this information (Figure 2). CPRs hold enormous potential for biological and medical discoveries, providing valuable insights into the diversity and complexity of life on Earth.

CPRs should be an essential target for future research to address microbial ecology problems, such as nutrient cycling competition, cross-feeding, community dynamics resulting from microbe predation, and antagonistic interactions between microorganisms (Figure 2). Their interactions with other organisms significantly impact various human activities, including agriculture, water treatment, etc. (Figure 2) [95]. The study of these ecological phenomena requires isolated strains, as direct observation of natural ecosystems is much more challenging due to the higher complexity and lack of experimental control. For instance, around half of the genes in most CPR genomes have no known function. This suggests that studying these microorganisms could yield significant opportunities for scientific discovery. As technology continues to improve, we can therefore expect to isolate these fastidious microbes and discover even more about the roles they play in their environments (Figure 2).

## Figures and Tables

**Table 1:** Comparison of the most common features of CPRs and classical bacteria.

General Characteristics	Candidate Phyla Radiation	Classical Bacteria
Genome size	Between 400 and 1500 kilobases	4.6 Megabase
Cell size	Between 100 and 300 nm	Between 0.5 and 3 μm
Cell shape	Coccoid	Different cell shapes
Lifestyle	Exo-symbiosis or parasitism	Free living
Multiplication strategy	Cell division	Cell division
Cultured microorganism	Not yet	Yes
Genes number	400 to 1500	4500
Cell membrane	Gram+	Gram+/Gram−
Copy of 16S rRNA	One	One or more
16S rRNA gene length	Between 1400 and 4400 bases	1500 bases
23S rRNA gene length	5000 bases	3000 bases
5S rRNA gene length	Between 107 and 117 bases	120 bases
Presence of insertion sequences in RNA	Yes	No
Average protein length	100 amino acids	300 amino acids
Hypothetical protein number	Hight	Low
Protein coding genes number	1000	5000

## Data Availability

Not applicable.
